# Review of Current Principles of the Diagnosis and Management of Brain Metastases

**DOI:** 10.3389/fonc.2022.857622

**Published:** 2022-05-24

**Authors:** Alex W. Brenner, Akash J. Patel

**Affiliations:** Department of Neurosurgery, Baylor College of Medicine, Houston, TX, United States

**Keywords:** metastasis, radiosurgery, laser interstitial thermal therapy (LITT), craniotomy, immunotherapy, whole brain radiotherapy (WBRT), SRS

## Abstract

Brain metastases are the most common intracranial tumors and are increasing in incidence as overall cancer survival improves. Diagnosis of brain metastases involves both clinical examination and magnetic resonance imaging. Treatment may involve a combination of surgery, radiotherapy, and systemic medical therapy depending on the patient’s neurologic status, performance status, and overall oncologic burden. Advances in these domains have substantially impacted the management of brain metastases and improved performance status and survival for some patients. Indications for surgery have expanded with improved patient selection, imaging, and intraoperative monitoring. Robust evidence supports the use of whole brain radiotherapy and stereotactic radiosurgery, for both standalone and adjuvant indications, in almost all patients. Lastly, while systemic medical therapy has historically provided little benefit, modern immunotherapeutic agents have demonstrated promise. Current investigation seeks to determine the utility of neoadjuvant radiotherapy and laser interstitial thermal therapy, which have shown benefit in limited studies to date. This article provides a review of the epidemiology, pathology, diagnosis, and treatment of brain metastases and the corresponding supporting evidence.

## Introduction

The incidence of brain metastases is difficult to quantify but is estimated to reach up to 100,000 new cases per year in the United States (1) and continues to increase due to improved diagnostic testing, increasing cancer survival, and an overall increase in life expectancy throughout the population irrespective of a diagnosis of cancer (2). Metastases are by far the most common brain tumors, accounting for over half of all intracranial neoplasms, and outnumber both malignant and benign primary brain tumors combined (2).

## Epidemiology

The most common primary source of brain metastases is lung cancer, accounting for over half of all instances; other prominent etiologies include breast cancer, melanoma, renal cell carcinoma, and colorectal cancer (2). These statistics largely reflect the relative incidence of primary cancers in general with the notable exception of prostate cancer, which, despite its distinction as the most common malignancy affecting men in the United states, rarely metastasizes to the brain (3).

Melanoma is the primary malignancy with the highest rate of metastasis to the brain, in up to 60% of cases (4), and while age of incidence varies by primary pathology, most brain metastases occur between the sixth to eight decades.

At the time of diagnosis, many patients—up to 85% based on radiographic and pathologic studies—are found to have multiple intracranial metastases; melanoma is also the most likely primary pathology to produce multiple lesions (5).

The incidence of brain metastases is also increasing due to advances in the efficacy of systemic therapy for cancer. For example, the advent of trastuzumab has dramatically improved survival in human epidermal growth factor receptor 2 (HER2)-positive breast cancer, leading to increasing incidence of brain metastases in patients as they live longer with systemic disease (6). A similar phenomenon has been observed in colorectal cancer; as brain involvement is a late-stage finding, and advances in systemic therapy have improved median survival nearly fourfold, the incidence of brain metastases has increased (7).

Brain metastases are both the most commonly occurring brain tumors and an increasingly common secondary complication of systemic cancers, and therefore warrant ever increasing clinical attention.

## Pathology

Brain metastases, like metastases in other organs, take on the histologic appearance of the primary pathology from which they arise. They most commonly spread *via* hematogenous dissemination to the junction of the gray and white matter of the brain, where changes in microvascular anatomy cause microemboli to be trapped. Metastatic tumors are more discrete and focal than primary glial neoplasms and tend to cause local displacement and compression of native brain tissue rather than diffuse infiltration. However, in some instances they may have infiltrating features, usually confined to within 5mm of the tumor capsule (2).

Between 80 and 85% of brain metastases are located in the cerebrum, with between 10 and 15% located in the cerebellum, and fewer than 5% in the brainstem (8).

## Diagnosis

The most common presenting signs and symptoms of brain metastases are those associated with any intracranial mass lesion, including headache, nausea, vomiting, focal neurologic deficits, seizure, and, in severe cases, disorders of consciousness. Prompt acquisition of cranial imaging is indicated. In an acute setting, computed tomography (CT) imaging can often be acquired more rapidly and may demonstrate the presence of a mass lesion, the extent of edema and mass effect on brain parenchyma, and the presence of tumor-associated hemorrhage. However, magnetic resonance imaging (MRI) is the gold standard diagnostic imaging modality in the evaluation of brain metastases. Metastases usually demonstrate contrast enhancement on T1 imaging and can have irregular internal appearance due to intralesional necrosis. As with other intracranial neoplasms, metastases often cause local vasogenic edema, which appears hyperintense on T2-weighted sequences and respects the borders of white matter tracts.

In patients with a symptomatic intracranial lesion and a known diagnosis of a primary cancer originating from outside the central nervous system, these imaging findings are highly suggestive of a diagnosis of brain metastasis (9); however, in patients without a history of cancer, a solitary brain mass is unlikely to be a metastatic lesion and is more probably a primary brain tumor. The presence of multiple lesions, especially when involving multiple intracranial compartments, is effectively diagnostic of metastasis.

With improved survival associated with advances in systemic oncologic therapies and increasing accessibility of advanced imaging, some brain metastases are diagnosed incidentally on imaging obtained for unrelated indications. Routine screening imaging of the brain is not necessary in patients with systemic cancer without neurologic symptoms.

## Treatment

Treatment modalities for brain metastases include surgery, radiation, and systemic therapy. Recent advances in all of these areas have resulted in improved survival of patients with brain metastases. Selection of treatment modalities depends on the size and location of the metastasis, the extent of intracranial and extracranial disease, and the patient’s performance status. Treatment paradigms are informed by consensus guidelines from several organizations, including the Congress of Neurological Surgeons (10–13) and the consortium of the American Society of Clinical Oncology, the Society for Neuro-Oncology, and the American Society for Radiation Oncology (14).

Initial treatment for symptomatic metastases includes dexamethasone to reduce edema (10), which can cause both focal neurologic deficits and increased intracranial pressure. In patients without a history of seizure, prophylactic antiepileptic medications are not indicated (11). In patients with altered mental status or abnormal vital signs, assessment of airway security and hemodynamic stability is of the highest priority.

### Systemic Medical Therapy

Historically, systemic cytotoxic chemotherapy has been ineffective in treating metastatic disease of the central nervous system both because these agents may be difficult to deliver across the blood-brain barrier and because patients with brain metastases usually present in advanced, sometimes treatment-resistant phases of disease.

However, the advent of modern small-molecule antineoplastic medications, such as systemic tyrosine kinase inhibitors (TKI), and immunotherapeutic monoclonal antibody agents, such as PD-1 and CTLA-4 inhibitors, has significantly changed the standard of care for many types of systemic cancer, including lung cancer, breast cancer, melanoma, and renal cell carcinoma, which are all among the most common malignancies to metastasize to the brain. Additionally, molecular and genetic subtyping of systemic malignancies has also allowed for increased precision in designing and delivering targeted therapy, which in some cases has been shown to be effective in treating brain metastases.

Immunotherapy has revolutionized the systemic treatment of some cancers to the extent that immunotherapy alone—without surgery or radiation therapy—is the preferred management for certain patients who present with brain metastases. In patients with melanoma and asymptomatic brain metastases, combination immunotherapy alone resulted in a three-year progression-free survival rate of 54% and an overall survival rate of 72%, without the need for radiation therapy or surgery (15, 16). Similarly, patients with HER-2 positive breast cancer and newly diagnosed, asymptomatic intracranial metastases may be offered targeted combination immunotherapy or TKI upfront as an alternative to radiation (17), although direct comparison data are limited. In patients with non-small cell lung cancer with ALK mutation, a variety of ALK-targeting TKI are considered first-line therapy and are FDA approved for both asymptomatic and symptomatic brain metastases without severe mass effect (18, 19), and furthermore, may even be offered as second-line agents for patients whose intracranial disease progresses on initial TKI therapy (20).

The viability of systemic treatment for patients with metastatic intracranial disease, including as both first- and second-line therapy without radiation or surgery, is a significant achievement in medical oncology in the modern era. However, its role in addressing acutely symptomatic, large lesions with risk for acute neurologic compromise is limited.

### Surgery

The role of surgery in the management of brain metastases has grown over time and is now considered the standard care for select patients. Resection of a metastatic lesion offers immediate relief of mass effect caused by the lesion, which can improve symptoms far more rapidly than radiation therapy to the same lesion. Additionally, resection of an edematous tumor can reduce the need for high-dose steroids in the short term, which may lead to reduction in steroid-related side effects.

Surgery also offers an opportunity for definitive histologic diagnosis, which radiation or systemic therapy alone does not. Although the majority of solitary brain tumors in patients with cancer are metastases from the known primary, in a minority of cases the tumor is a primary brain tumor, metastasis of a second systemic malignancy, or even a non-neoplastic lesion (9). Histopathologic diagnosis in these cases may inform further treatment, though surgery for biopsy and diagnostic confirmation alone is rarely indicated.

Patients with a single brain metastasis and a good overall preoperative performance status, generally measured as a score of > 70 by the Karnofsky Performance Scale (KPS), may be candidates for surgery to attain local control of metastatic disease. Evidence from randomized controlled trials suggests that surgery does not offer a benefit over radiotherapy when patients are not selected based on performance status and systemic disease burden (21). Patient selection for resection requires consideration of age, KPS, and the extent of systemic disease. These factors were evaluated by recursive partitioning analysis (RPA) in a landmark analysis which established RPA classes for patient selection, demonstrating that RPA class 1 patients—with age under 65, KPS of at least 70, and controlled primary disease without additional sites of metastasis—are most likely to benefit from surgery (22). In appropriately selected patients with tumor size > 2.5cm, resection carries a benefit in overall survival and local control over whole-brain radiation alone as demonstrated in studies of both disease-specific cohorts and all patients with oligometastatic disease (9, 23–26). Resection should be considered first-line means of local control for metastatic lesions > 3cm in diameter, as lesions of this size are less responsive to radiotherapy regardless of modality.

The decision to offer surgery must take into account the likelihood of the patient incurring a neurologic deficit. With modern microsurgical techniques and technology including frameless stereotactic navigation, intraoperative ultrasound, and intraoperative neuromonitoring (including awake and asleep mapping and stimulation), the risk of surgical morbidity for anatomically resectable lesions is low. However, not all metastatic lesions are amenable to surgery. Tumors located in deep nuclei and white matter tracts including the brainstem, thalamus, and basal ganglia are usually not considered resectable due to the risk of morbidity. In patients with cancer, avoiding a postoperative neurologic deficit is of utmost importance as patients with impaired performance status may not be offered further disease-directed therapy.

Surgical technique has also been shown to affect outcomes. Multiple studies have demonstrated that en bloc resection is superior to piecemeal resection both for oncologic benefit and perioperative morbidity. En bloc resection has been shown to decrease risk of leptomeningeal dissemination in both supratentorial and infratentorial metastases compared to piecemeal resection and even stereotactic radiosurgery (SRS) (27, 28) and is also associated with lower risk of local recurrence prior to adjuvant radiotherapy (29). A retrospective study evaluating postoperative complications based on surgical technique found that en bloc resection was associated with a lower rate of complications than piecemeal resection (30).

Advances in radiotherapy and systemic therapy have led to an expanded role for surgery in patients with multiple brain metastases and created a new indication for surgery in patients with symptomatic brain metastases: resection to reduce steroid dependence. Since dexamethasone interferes with the mechanism of action of certain types of modern immunotherapy, removing the edematous focus can facilitate rapid weaning of steroids and allow patients to resume systemic treatment with these highly effective agents. In these instances, resection of highly edematous lesions, even if smaller than 2cm and/or in patients with many intracranial metastases for which radiation would otherwise be the preferred treatment modality, may be offered for systemic oncologic benefit.

In modern neurosurgical practice, craniotomy for resection is not the only option for surgical management of brain metastases. Laser interstitial thermal therapy (LITT) involves magnetic resonance-guided thermal ablation of tissue *via* a relatively less invasive surgical approach than open craniotomy. Although ablation does not immediately address the mass effect of lesions requiring surgical resection, studies have demonstrated it is effective in achieving local control of recurrent previously irradiated brain metastases and reducing steroid requirement (31). Furthermore, LITT can be considered as a surgical alternative to craniotomy for local control for lesions that are anatomically unresectable due to the risk of neurologic morbidity (32). In patients with advanced disease and metastases which progress to recurrence or radiation necrosis following radiotherapy, LITT has been shown to prevent worsening of KPS, reduce steroid requirement, and preserve quality of life and cognitive function over 12 weeks postoperatively (33). Therefore, LITT may be an increasingly appropriate palliative option for patients with advanced disease who may not be able to tolerate craniotomy, or who may not benefit from open surgery in light of their systemic disease burden and limited life expectancy. However, its availability is limited by operator expertise and availability of advanced laser and intraoperative thermosensitive MR technology.

### Radiation Therapy

Radiation therapy is the mainstay of treatment of intracranial metastases to attain local control and prevent growth and recurrence. Several modalities of radiation therapy are used in modern practice, both alone and in conjunction with surgery. The main modalities are whole brain radiation therapy (WBRT), stereotactic radiosurgery (SRS), and, more recently, brachytherapy.

Whole brain radiation therapy has been used to treat intracranial metastases for nearly 70 years. Advantages of this modality include treating the entire brain, providing therapy to all sites of metastasis, including microscopic deposits potentially not identifiable on imaging. For this reason, prophylactic WBRT is offered to many patients with small cell lung cancer, which characteristically metastasizes to the brain early in its clinical course; multiple studies have supported this indication even in patients with no clinical or radiographic evidence of intracranial disease (34, 35). Additionally, it is the easiest modality to deliver to patients who may not tolerate the placement of a stereotactic headframe for radiosurgery.

However, WBRT is inherently imprecise and targets both malignant and normal brain tissue. This results in a higher risk of deleterious neurologic side effects, including memory and cognitive deficits in the long term, and headache, nausea, and vomiting in the short term. Most patients who receive WBRT should be prescribed memantine to mitigate neurologic side effects (36). Additionally, for patients with metastases not involving the hippocampus, hippocampal-sparing WBRT offers improved cognitive outcomes compared to conventional WBRT (37, 38).

Due to the potential for delayed neurologic injury, WBRT is best suited to patients with a relatively shorter life expectancy, which coincides with its suitability for patients with multiple intracranial lesions. Neurologic injury is associated with higher fractionated doses; therefore, a paradigm of smaller doses in more fractions has been shown to be superior (12). A standard regimen may include 30 Gy in 10 or 15 fractions; alterations in dose and fractionation do not result in improvement in survival or local control, which has been established *via* multiple randomized controlled trials (39, 40).

In many patients with extensive intracranial disease burden, prognosis is poor despite treatment with WBRT. Therefore, symptomatic treatment with steroids and supportive care is also an option for some of these patients, especially those with low performance status; a randomized controlled trial demonstrated that optimal supportive care was non-inferior to optimal supportive care with WBRT in patients with non-small cell lung cancer in terms of overall survival and quality of life (41).

WBRT can also be used in an adjuvant role to prevent local recurrence after surgery and further distant metastasis. Adjuvant WBRT has been shown to reduce the risk of both local recurrence and distant metastasis by more than half but does not affect overall survival or functional independence in this population (42, 43).

Stereotactic radiosurgery (SRS) has emerged as a favorable alternative to WBRT in many patients with brain metastases. SRS delivers radiation to discrete sites at the intersection of highly collimated sources, resulting in high doses at the site of intersection with rapid falloff in the delivered dose away from the target. It therefore carries the advantage of effective treatment to discrete lesions without the harmful off-target effects associated with WBRT but is less effective in treating many lesions. However, linear accelerator-based stereotactic radiotherapy has recently emerged as an option for the targeted treatment of multiple brain metastases in fractionated doses (44, 45).

SRS is the preferred first-line treatment for patients with oligometastatic disease and lesions < 3cm in maximal diameter without acutely life-threatening presentation, especially when located in an eloquent area or when surgical resection may otherwise result in neurologic deficit. Current guidelines suggest that patients with up to 4 metastases, and with >4 metastases if the total tumor volume is < 7cc, should be treated with SRS upfront instead of WBRT (13). Although some evidence exists for improved intracranial disease control with SRS plus WBRT for oligometastatic disease (46), more recent randomized trials have shown that SRS plus WBRT carries no advantage in overall survival compared to SRS alone and is associated with greater neurocognitive morbidity (43, 47, 48).

SRS has also been established as a mainstay of adjuvant radiotherapy in patients with surgical metastatic disease. Current neurosurgical guidelines recommend consideration of SRS to the postoperative cavity following resection of a solitary metastasis based on studies demonstrating effective local control and decreased morbidity compared to adjuvant WBRT (49–51). Another recent randomized trial demonstrated that adjuvant SRS in patients with up to three resected metastases significantly lowered the rate of local recurrence, suggesting that, even for patients with multiple resected lesions, SRS may be an effective alternative to WBRT (52).

More recently, investigators have explored the role for neoadjuvant SRS in the treatment of oligometastatic disease. While no clinical trial to date has evaluated the efficacy of SRS in the neoadjuvant role (53), one combination prospective/retrospective study has suggested that it is safe and does not increase the risk of radiation necrosis or leptomeningeal spread of disease (54); further studies are currently underway (55, 56).

Brachytherapy is the third modality of radiation therapy employed in the treatment of brain metastases. Conceptually, the implantation of radioactive source material into the tumor cavity at the time of surgical resection dates back to the 1930s and the early age of neurologic surgery, but has been limited by the danger of systemic toxicity of indwelling radioisotopes to both the patient and bystanders (57). However, more recent advances in bioabsorbable materials science and the use of ^125^I and especially ^131^Cs offer the possibility of delivering high-dose radiotherapy to disease sites with limited off-target effects in a safe biodegradable delivery system. Early trials have demonstrated safety and efficacy in limited applications (58–60).

## Discussion

Metastasis to the brain is a frequent complication of many of the most common types of systemic malignancies. Over the last generation, dramatic advancements in systemic medical oncology, surgical technique and technology, and radiation therapy have resulted in both a dramatic increase in the incidence of brain metastases—due to increased overall cancer survival—and a dramatic improvement in the options available to neurosurgeons, radiation oncologists, and medical oncologists in their management.

Future advancements in the treatment of brain metastases may emerge from cutting-edge fields including genomics, radiomics, and artificial intelligence. Basic science and translational studies have identified mutations and epigenetic factors that drive expression of brain metastases in animal models (61, 62), which may present an option for targeted therapy in the future. As imaging technology continues to evolve, radiologic characteristics of brain metastases are being evaluated for their utility as non-invasive biomarkers that may guide prognosis and treatment (63). Machine learning may further be able to incorporate radiologic (64, 65), genomic, and clinical characteristics of individual patients to offer more precise and individualized diagnosis and treatment options than consensus-based guidelines can offer.

The nature of metastatic cancer renders each patient with brain metastases unique, and therefore no single treatment paradigm is appropriate for every such patient. Patients with oligometastatic disease and good functional status benefit most from surgical resection with adjuvant stereotactic radiosurgery and medical therapy where indicated. Patients with widespread metastatic disease still benefit most from palliative whole brain radiation therapy to achieve maximal local control and prevent neurologic worsening. For patients whose disease burden falls in between these extremes, a variety of treatment modalities are available with various levels of supporting evidence. Ultimately, each patient’s optimal treatment paradigm must be developed in collaboration between the neurosurgeon, the radiation oncologist, the medical oncologist, and the patient. As further advances in systemic therapy, neurosurgical technique, and radiation therapy are achieved, more options will be available to treat patients with metastatic cancer involving the brain.

## Author Contributions

AB – First author. AP – Senior author. All authors contributed to the article and approved the submitted version.

## Conflict of Interest

The authors declare that the research was conducted in the absence of any commercial or financial relationships that could be construed as a potential conflict of interest.

## Publisher’s Note

All claims expressed in this article are solely those of the authors and do not necessarily represent those of their affiliated organizations, or those of the publisher, the editors and the reviewers. Any product that may be evaluated in this article, or claim that may be made by its manufacturer, is not guaranteed or endorsed by the publisher.
